# Micro-tomographic characterization of the root and canal system morphology of mandibular first premolars in a Chilean population

**DOI:** 10.1038/s41598-020-80046-1

**Published:** 2021-01-08

**Authors:** Alfredo Sierra-Cristancho, Luis González-Osuna, Daniela Poblete, Emilio A. Cafferata, Paola Carvajal, Carla P. Lozano, Rolando Vernal

**Affiliations:** 1 Periodontal Biology Laboratory, Faculty of Dentistry, Universidad de Chile, Santiago, Chile; 2 Faculty of Dentistry, Universidad Andres Bello, Santiago, Chile; 3 Department of Periodontology, School of Dentistry, Universidad Científica del Sur, Lima, Perú; 4 Department of Conservative Dentistry, Faculty of Dentistry, Universidad de Chile, Santiago, Chile; 5 Oral Biology and Biochemistry Laboratory, Institute for Research in Dental Sciences, Faculty of Dentistry, Universidad de Chile, Santiago, Chile

**Keywords:** Oral anatomy, Dental diseases

## Abstract

This study aimed to analyze the root anatomy and root canal system morphology of mandibular first premolars in a Chilean population. 186 teeth were scanned using micro-computed tomography and reconstructed three-dimensionally. The root canal system morphology was classified using both Vertucci’s and Ahmed’s criteria. The radicular grooves were categorized using the ASUDAS system, and the presence of Tomes’ anomalous root was associated with Ahmed’s score. A single root canal was identified in 65.05% of teeth, being configuration type I according to Vertucci’s criteria and code ^1^MP^1^ according to Ahmed’s criteria. Radicular grooves were observed in 39.25% of teeth. The ASUDAS scores for radicular grooves were 60.75%, 13.98%, 12.36%, 10.22%, 2.15%, and 0.54%, from grade 0 to grade 5, respectively. The presence of Tomes’ anomalous root was identified only in teeth with multiple root canals, and it was more frequently associated with code ^1^MP^1–2^ of Ahmed’s criteria. The root canal system morphology of mandibular first premolars showed a wide range of anatomical variations in the Chilean population. Teeth with multiple root canals had a higher incidence of radicular grooves, which were closely related to more complex internal anatomy. Only teeth with multiple root canals presented Tomes’ anomalous root.

## Introduction

The human teeth have a wide variability of shapes and configurations, which are directly related to the genetic determinants, ethnicity, and geographical origin of individuals^1,2^. In this context, comprehensive knowledge of the tooth anatomy and root canal system morphology is essential to achieve successful root canal debriding, shaping, and filling during endodontic treatment^3^. Indeed, a poor understanding of the three-dimensional (3D) root canal system morphology can result in the inability to identify and treat all the root canals; thus, compromising the total pulp removal and complete disinfection and finally, leading to endodontic treatment failure^4^.

An ongoing controversial discussion about the external and internal anatomy of the mandibular first premolar can be found in the scientific literature^5–9^. For a better understanding of the internal configuration of the root canal system, Vertucci’s score has been widely used to analyze anatomic variations^10^. This system allows us to categorize the configuration of the root canals; however, it is not designed to describe the number and configuration of the roots. In this sense, Ahmed et al*.*^11^ proposed a new classification system, which provides more information about the number of roots, the anatomy of the main and accessory root canals^12^, and the potential anomalies in the root canal system morphology^13^. Regarding the external configuration, radicular grooves are frequently observed on the mesial or distal surfaces of the root of mandibular first premolars, and they are often associated with complex internal anatomical features, including C-shaped canals or isthmus, among other root configurations^14^. Tomes’ anomalous root is included within these external roots anatomy variations and, according to the Arizona State University Dental Anthropology Scoring System (ASUDAS), its severity can be graded from 0 to 5, being the presence of Tomes’ roots equivalent to grades 3–5^15^. To date, limited research on radicular grooves of mandibular first premolars has been reported^5–9^ and, to the best of our knowledge, there are no studies using Ahmed’s criteria and ASUDAS scoring to describe mandibular first premolars in the Chilean population.

Different methodologies have been used to study the internal and external anatomy of the teeth, such as conventional and digital radiography, clearing techniques, cross-sectioning, scanning electron microscopy, and stereomicroscope^4,16–18^. Nowadays, micro-computed tomography (micro-CT) is considered the gold standard for anatomical studies due to it being a non-invasive and reproducible technique that allows a qualitative and quantitative 3D-characterization of the teeth morphology^19,20^. Therefore, this study aimed to analyze the root anatomy and canal system morphology of mandibular first premolars obtained from a Chilean population using micro-CT. Internal morphology was analyzed using Vertucci’s and Ahmed’s criteria, and external morphology was analyzed using the ASUDAS scoring. The relationship between ASUDAS scoring, in particular the presence of Tomes’ anomalous root, and the complexity of the root canal system morphology scored with Ahmed’s criteria were also explored.

## Methods

### Teeth selection and preparation

A selected sample of human mandibular first premolars from a Chilean population (n = 186), extracted for orthodontic reasons, was utilized in this study. Selection criteria were as follows: Teeth with fully formed apices, intact crowns (i.e., no carious lesions, no fractures, no restorations, and no endodontic treatment), and absence of root resorption, root caries, or root fractures. Sampled teeth were cleaned by immersion in 5% sodium hypochlorite for 30 min and reserved in 10% neutral buffered formalin, before being debrided of remnants of periodontal tissues and dental calculus by using an ultrasonic scaler (NSK, Tokyo, Japan). Then, the specimens were stored in a moisturizing solution at room temperature until further analysis. The study (Protocol #2018/03) was approved by the Ethics Committee for Human Research of the Faculty of Dentistry from Universidad de Chile. All the participants agreed to participate in the study by signing an institutional review board-approved informed consent. The research protocol was carried out in full accordance with the ethical principles of the Declaration of Helsinki of the World Medical Association, 2008.

### Micro-CT scanning and three-dimensional reconstruction

All teeth were scanned using a high-resolution micro-CT device (SkyScan 1278, Bruker, Kontich, Belgium) at 65 kV, 692 mA, with a rotation step of 0.2°, 360° around the vertical axis, and 50 µm voxel size, using a 1-mm-thick aluminum filter. The acquired images were reconstructed in a 3D-dataset using the NRecon v.1.6.9 software (Bruker, Kontich, Belgium). The 3D-model was adjusted to visualize the internal (opaque) and external (transparent) tooth structures. The segmentation of the structures was obtained using the CTAn v.1.12 software (Bruker, Kontich, Belgium).

### Morphological analysis

**Figure 1:**
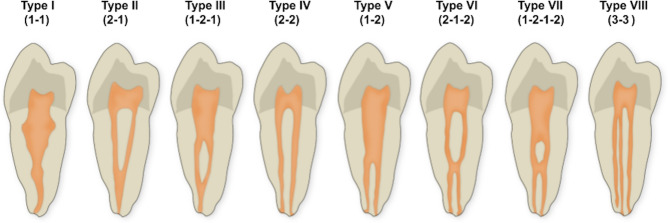
Representative illustrations of the different configuration types of the root canal system morphology according to Vertucci’s criteria.

**Figure 2:**
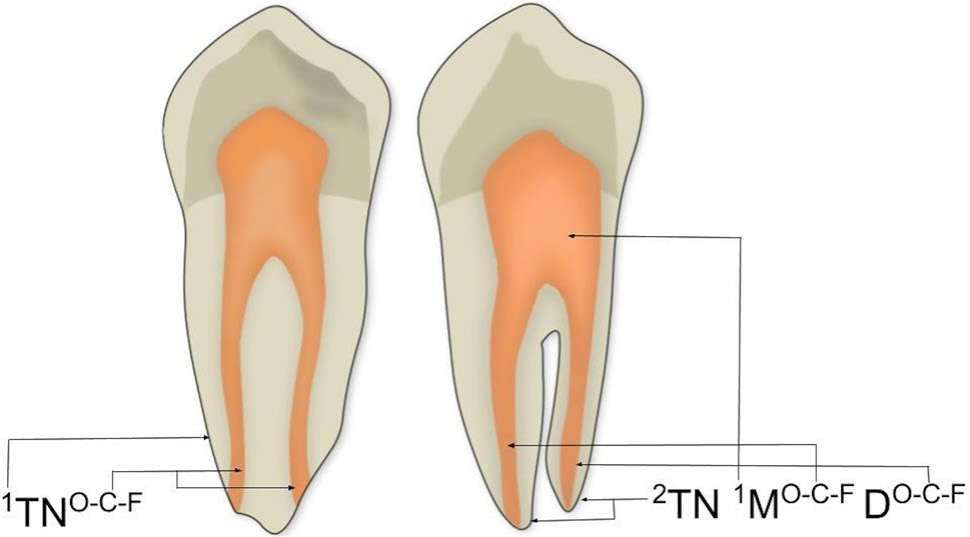
Representative illustrations of the root canal system morphology coded according to Ahmed’s criteria. The superscript on the left of TN corresponds to the number of roots. The superscript on the right of TN, in a single-rooted tooth, corresponds to the root canal configuration type, and in a double-rooted tooth, corresponds to the root canal configuration type on the right of each root, being M: mesial and D: distal. Whether the root has a common configuration coronal to canal bifurcation, then the canal configuration common to both root is written before the identification of the roots. The same code applies to multi-rooted teeth. O–C–F corresponds to the root canal configuration type starting from the orifice, passing through the canal to the foramen. *TN* tooth number.

Two previously calibrated examiners (A.S-C. and L.G-O.) individually analyzed the reconstructed images and, in case of interpretation disagreements, the discrepancies were resolved by consensus. The DataViewer v.1.4.4 and CTVol v.2.2.10 softwares (Bruker, Kontich, Belgium) were used to evaluate the following characteristics:

1. Type of configuration of root canals, according to Vertucci’s and Ahmed’s criteria. Figure 1 depicts the different configuration types of the root canal system according to Vertucci’s criteria. Figure 2 depicts the different codes used to characterize the root canal system morphology according to Ahmed’s criteria. Ahmed’s criteria coding for single-rooted, double-rooted, and multi-rooted teeth are shown in Table 1.

**Table 1:** Ahmed’s criteria coding assigned to single, double, or multiple root teeth.

Tooth type	Code
Single-rooted	^1^TN^O–C–F^
Double-rooted	^2^TN R1^O–C–F^ R2^O–C–F^
Multi-rooted	^n^TN R1^O–C–F^ R2^O–C–F^ Rn^O–C–F^

*C* canal; *F* foramen; n, refers to three or more roots; *O* orifice; *R* root; *TN* tooth number.

**Figure 3:**
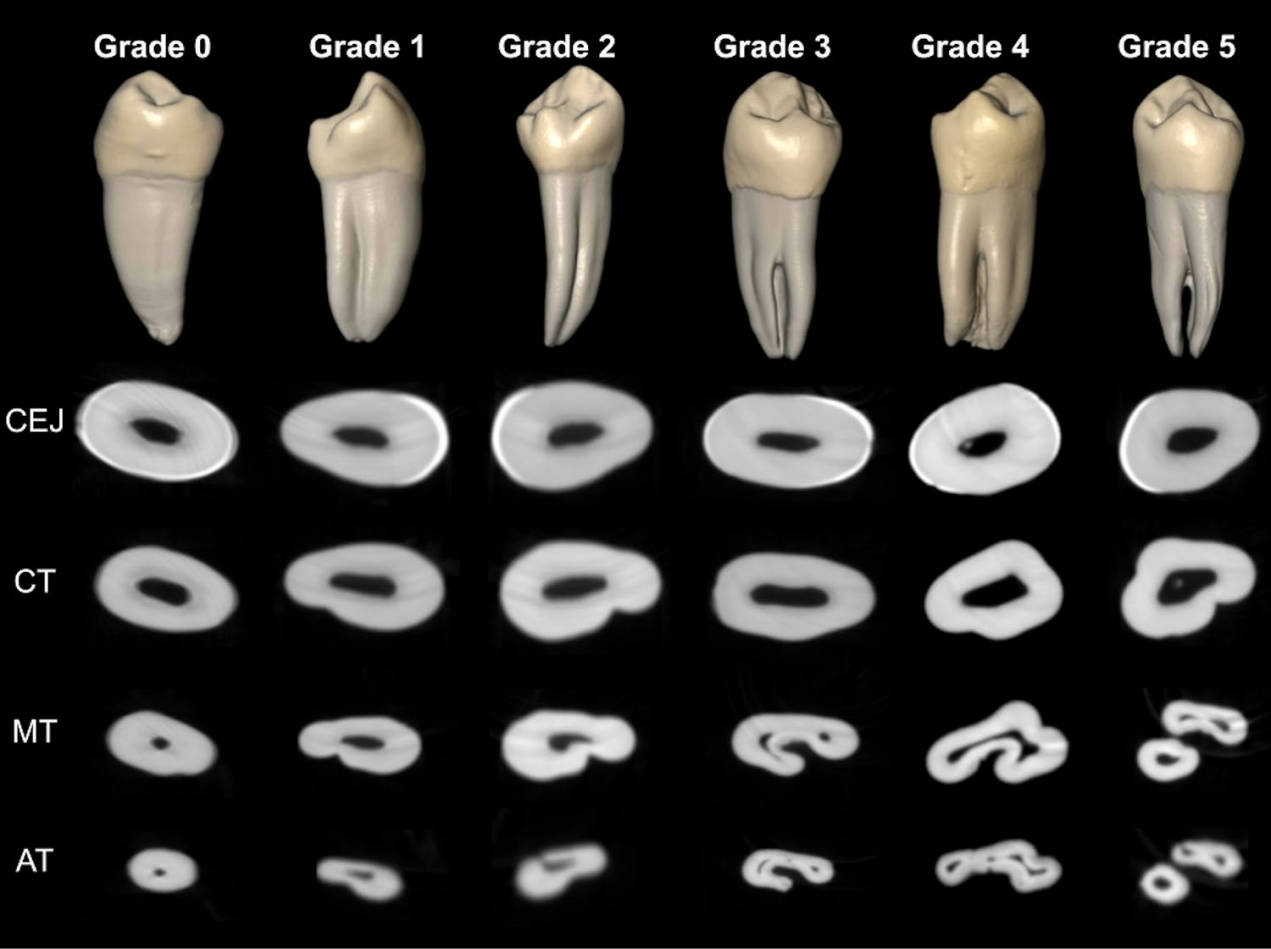
Micro-CT images showing the radicular grooves classified according to ASUDAS scoring. *AT* apical third; *CEJ* cementoenamel junction; *CT* cervical third; *MT* middle third (Images obtained using the CTAn v.1.12 software, Bruker).

2. Presence and categorization of radicular grooves according to ASUDAS scoring. As shown in Fig. 3, the radicular groove scores were as follows: Grade 0: A radicular groove is absent, or if present, it is shallow with rounded indentation. Grade 1: A radicular groove is present and has a shallow V-shaped cross-section. Grade 2: A radicular groove is present and has a moderately deep V-shaped cross-section. Grade 3: A radicular groove is present and has a markedly deep V-shaped cross-section, such that the radicular groove extends to, at least, $\frac{1}{3}$ of the total root length. Grade 4: A radicular groove is deeply invaginated on both the mesial and distal root surfaces. Grade 5: Two independent roots are present, such that their length is at least $\frac{1}{4}-\frac{1}{3}$ of the total root length. In order to analyze the relationship between the presence of Tomes’ anomalous root and the complexity of the root canal system morphology, scored with Ahmed’s criteria, the analyzed teeth were classified into two groups: the non-Tomes’ root group, corresponding to grade 0 to grade 2 of ASUDAS scoring, and the Tomes’ root group, corresponding to grade 3 to grade 5 of ASUDAS scoring.

3. Anatomical characteristics, such as number and location of apical foramina, frequency of accessory canals, and presence of C-shaped canals, isthmus, or apical deltas.

### Statistical analysis

Descriptive data regarding roots’ anatomical features were presented as frequencies and cumulative percentages. The potential association between the presence of Tomes’ anomalous root and the internal complexity of the root canal system, according to Ahmed’s criteria, was analyzed using the Chi-square test. A p-value of < 0.05 was considered statistically significant. Data analysis was performed using the statistics software SPSS v.22.0 (IBM Corp, Armonk, NY, USA).

## Results

### Root canal system morphology

**Figure 4:**
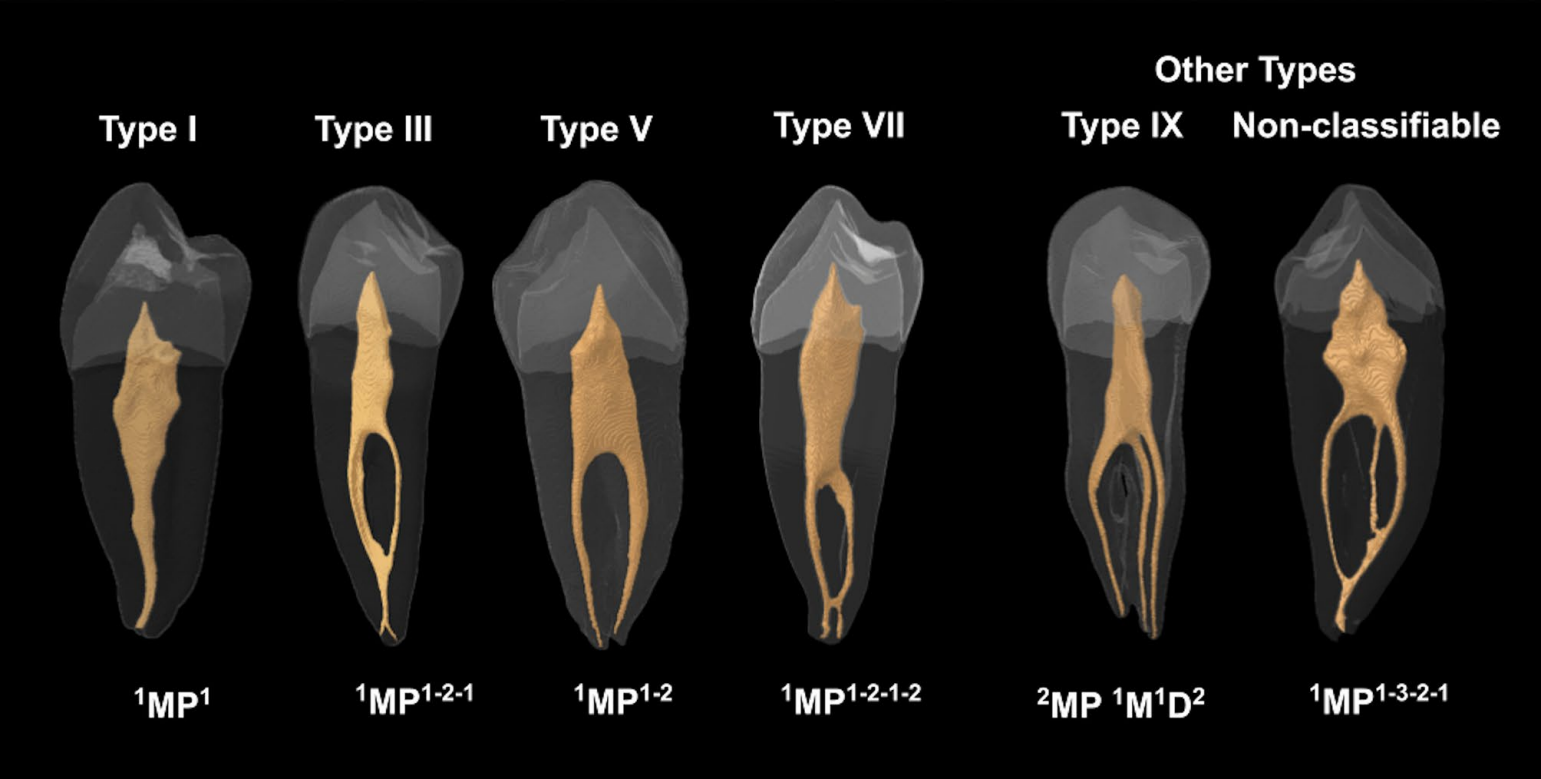
Micro-CT images showing the root canal configuration of the analyzed teeth using Vertucci’s criteria (above) and Ahmed’s criteria (below). *D* distal; *M* mesial; *MP* mandibular first premolar (Images obtained using the CTAn v.1.12 software, Bruker).

The root canal system morphology of mandibular first premolars showed a wide range of anatomical variations in the Chilean population (Table 2 and Fig. 4). According to Vertucci’s criteria, the most common configuration was the type I (1–1) in 65.05% of the analyzed teeth, followed by type V (1–2) in 24.19% of the samples. The less frequent configuration was the type VII (1–2–1–2) in 2.15% of teeth. The configurations type II (2–1), IV (2,2), and VI (2–1–2) were not observed, and 6 teeth showed other types of configuration not included in Vertucci’s criteria. These other types of configuration are depicted in Fig. 4 and correspond to 2.69% for configuration 1–3 and 0.54% for configuration 1–3–2–1. According to Ahmed’s criteria, the code ^1^MP^1^ was the most prevalent in single-rooted teeth, occurring in 65.05% of the samples, followed by code ^1^MP^1–2^ in 24.19% of teeth. The less frequent code was the ^1^MP^1–3–2–1^ in 0.54% of the analyzed teeth, and codes ^1^MP^2–1^, ^1^MP^2–2^, and ^1^MP^2–1–2^ were not identified. The unique double rooted-tooth included in the study had three root canals and was classified with the code ^2^MP ^1^M^1^D^2^.

**Table 2:** Root canal system morphology of the analyzed mandibular first premolars according to Vertucci’s criteria and their correspondence with Ahmed’s criteria. *D* distal; *M* mesial; *MP* mandibular first premolar.

Vertucci’s criteria				Ahmed’s criteria		
Type	Canal pattern	*N*	(%)	Code	*N*	(%)
				Single-rooted		
Type I	(1–1)	121	(65.05)	^1^MP^1^	121	(65.05)
Type II	(2–1)	0	(0.0)	^1^MP^2–1^	0	(0.0)
Type III	(1–2–1)	10	(5.38)	^1^MP^1–2–1^	10	(5.38)
Type IV	(2–2)	0	(0.0)	^1^MP^2–2^	0	(0.0)
Type V	(1–2)	45	(24.19)	^1^MP^1–2^	45	(24.19)
Type VI	(2–1–2)	0	(0.0)	^1^MP^2–1–2^	0	(0.0)
Type VII	(1–2–1–2)	4	(2.15)	^1^MP^1–2–1–2^	4	(2.15)
Type VIII	(3–3)	0	(0.0)	^1^MP^3–3^	0	(0.0)
Other types				^1^MP^1–3^	4	(2.15)
Type IX	(1–3)	5	(2.69)	^1^MP^1–3–2–1^	1	(0.54)
Non-Classifiable	(1–3–2–1)	1	(0.54)	Double-rooted		
				^2^MP ^1^M^1^D^2^	1	(0.54)

### Radicular grooves

According to the ASUDAS scoring (Table 3), 60.75% of teeth did not present a radicular groove (grade 0). Among teeth showing radicular grooves, the scores were 13.98%, 12.36%, 10.22%, and 2.15%, respectively, from grade 1 to grade 4. Only a tooth had radicular groove grade 5.

**Table 3:** Radicular groove type observed in mandibular first premolars according to the ASUDAS scoring.

ASUDAS score	N	%
Grade 0	113	60.75
Grade 1	26	13.98
Grade 2	23	12.36
Grade 3	19	10.22
Grade 4	4	2.15
Grade 5	1	0.54

The relation between the radicular groove, categorized using the ASUDAS scoring, and the root canal system morphology, scored using Ahmed’s criteria, is detailed in Table 4. Among the teeth with a single root canal, 65.05% presented the code ^1^MP^1^, being all of them grades 0–2 according to the ASUDAS scoring. 34.95% of teeth had multiple root canals, being 41 teeth grades 0–2, and 24 teeth grades 3–5 (Tomes’ anomalous root), according to the ASUDAS scoring. The code ^1^MP^1–2^ was the most common pattern in teeth with multiple root canals, occurring in 24.19% of the analyzed teeth. From them, 26 teeth were grades 0–2, and 19 teeth were grades 3–5 (Tomes’ anomalous root), according to the ASUDAS scoring. In teeth with other Ahmed’s codes, 15 teeth were grades 0–2, and 5 teeth were grades 3–5 (Tomes’ anomalous root), according to the ASUDAS scoring. From the analyzed teeth, 162 teeth were non-Tomes’ root, and most of them (74.69%) were teeth with a single root canal (*P* < 0.001). The presence of Tomes’ anomalous root was identified only in teeth with multiple root canals, and most of them (79.17%) were code ^1^MP^1–2^ of Ahmed’s criteria (*P* = 0.031).

**Table 4:** Relation between the radicular groove categorized using the ASUDAS scoring and the root canal system morphology scored using Ahmed’s criteria.

ASUDAS Score	N	Single root canal		Multiple root canals					
		Code ^1^MP^1^ (%)		Total (%)		Code ^1^MP^1–2^ (%)		Other Codes (%)	
ASUDAS 0	113	105	(92.92)	8	(7.08)	6	(5.31)	2^a^	(1.77)
ASUDAS 1	26	15	(57.69)	11	(42.31)	10	(38.46)	1^b^	(3.85)
ASUDAS 2	23	1	(4.35)	22	(95.65)	10	(43.48)	12^c^	(52.17)
ASUDAS 3	19	0	(0.0)	19	(100)	17	(89.47)	2^d^	(10.53)
ASUDAS 4	4	0	(0.0)	4	(100)	2	(50.00)	2^e^	(50.00)
ASUDAS 5	1	0	(0.0)	1	(100)	0	(0.0)	1^f^	(4.76)
Total	186	121	(65.05)	65	(34.95)	45	(24.19)	20	(10.75)
Non-Tomes’ root	162 (100%)	121	(74.69)*	41	(25.31)	26	(16.05)	15	(9.26)
Tomes’ root	24 (100%)	0	(0.0)	24	(100)	19	(79.17)*	5	(20.83)

^a^One tooth with code ^1^MP^1–2–1^ and one tooth with code ^1^MP^1–3^.

^b^One tooth with code ^1^MP^1–3^.

^c^Eight teeth with code ^1^MP^1–2–1^ and four teeth with code ^1^MP^1–2–1–2^.

^d^One tooth with code ^1^MP^1–2–1^ and one tooth with code ^1^MP^1–3–2–1^.

^e^Two teeth with code ^1^MP^1–3^.

^f^One tooth with code ^2^MP^1^ M^1^DB^1^DL^1^.

*P < 0.05.

### Anatomical features

**Figure 5:**
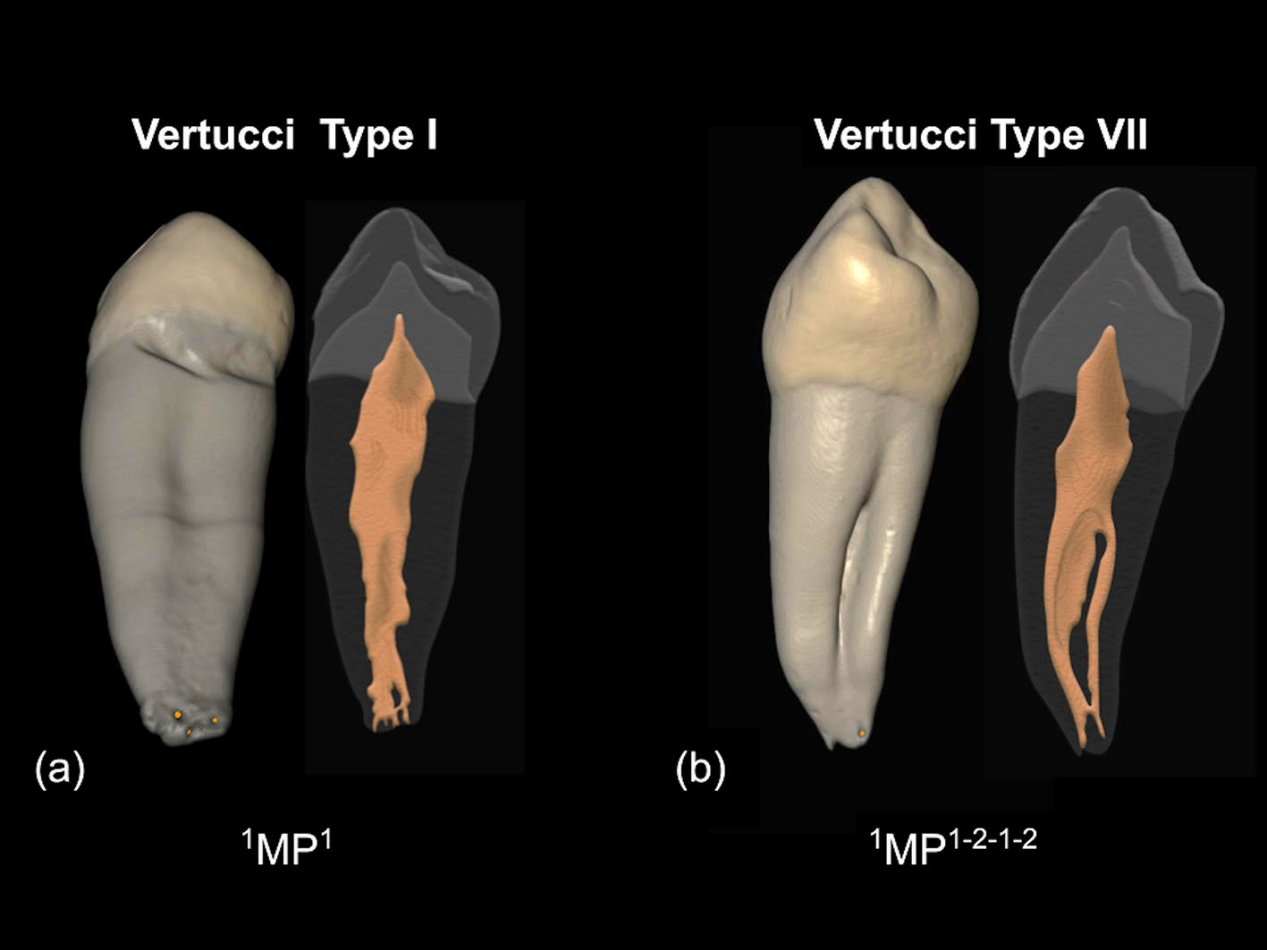
Micro-CT images showing the external and internal anatomical features analyzed in mandibular first premolars. (**a**) Non-Tomes' root (ASUDAS grade 0), containing a single root canal (above, Vertucci’s classification Type I; below, Ahmed’s criteria ^1^MP^1^) and the presence of apical delta and multiple apical foramina in the apical third of the root. (**b**) Tomes' root (ASUDAS grade 3), containing multiple root canals (above, Vertucci’s classification Type VII; below, Ahmed’s criteria ^1^MP^1–2–1–2^) and the presence of C-shaped canal in the middle third of the root. *MP* mandibular first premolar (Images obtained using the CTAn v.1.12 software, Bruker).

Table 5 shows the general anatomical characteristics of the 186 mandibular first premolars analyzed in the present study. 99.46% of teeth possessed a single root, and only a tooth had two roots. In the apical region, a single apical foramen was found in 36.56% of the analyzed teeth, followed by two apical foramina in 27.42% of teeth, and the apical delta was observed in 26.88% of the analyzed teeth (Fig. 5a). The most frequent location of the apical foramina was lateral, identified in 62.37% of the analyzed teeth. 62.90% of teeth presented an accessory canal, being the most frequent location (43.55%) in the apical third of the root. 29.57% of teeth had C-shaped canals, being the most frequent localization (19.35%) in both the middle and apical thirds of the root (Fig. 5b). Besides, 31.72% of the teeth had isthmus.

**Table 5:** General anatomical features of the analyzed teeth.

Feature	N	%
**Number of roots**		
1	185	99.46
2	1	0.54
**Number of apical foramina**		
1	68	36.56
2	51	27.42
3	29	15.59
4	38	20.43
**Location of the apical foramina**		
Central	70	37.63
Lateral	116	62.37
**Accessory canal**		
Coronal third	0	0.00
Middle third	13	6.99
Apical third	81	43.55
Both middle and apical thirds	23	12.37
**C-shaped**		
Coronal third	0	0.00
Middle third	13	6.99
Apical third	6	3.23
Both middle and apical thirds	36	19.35
**Isthmus**		
Coronal third	0	0.00
Middle third	36	19.35
Apical third	18	9.68
Both middle and apical thirds	5	2.69
Apical delta	50	26.88

## Discussion

Comprehensive knowledge of the root canal system morphology is crucial for successful endodontic treatment^3^. The mandibular first premolar may possess one or more root canals with many configuration patterns; however, dentists generally look for a single root and a single canal^6,9,18^. In this study, the root canal system morphology of mandibular first premolars showed highly variable and complex anatomy in the Chilean population. Teeth with multiple root canals had a more complex root canal configuration and a higher frequency of radicular grooves compared with teeth with a single root canal. Indeed, teeth with multiple root canals frequently possess C-shaped canals, isthmus, accessory channels, and apical deltas. Furthermore, a direct relationship between the presence of Tomes’ anomalous root and the internal anatomy of the analyzed teeth was observed.

Vertucci’s classification allowed the identification of four traditional types of root canal configuration in 180 of the analyzed teeth, being the type I the most prevalent (65.05%). Besides, a supplemental configuration type IX^21^ was detected in 5 teeth (2.69%), and a configuration Vertucci non-classifiable (1–3–2–1) was detected in 1 tooth (0.54%). A single canal was identified in 65.05% of teeth, and multiple canals were identified in 34.95% of teeth, which is consistent with previous studies in Chinese populations. Liu et al*.*^5^ reported single canal detection in 65.2% of teeth and multiple canal detection in 26.1% of the analyzed teeth, while Dou et al*.*^8^ reported 64.04% and 35.96%, respectively. Similarly, Alkaabi et al*.*^22^ reported the single canal detection in 68% of teeth and multiple canal detection in 32% of teeth in an Emirati population. Conversely, Pedemonte et al*.*^18^ reported a different detection in the Chilean population, with an incidence of multiple root canal in 16.83% of the analyzed teeth. The discrepancy with the herein presented data could be explained by the different methods used for teeth analysis. In the present study, we use the micro-CT technology in order to ensure high visibility of anatomic structures, while Pedemonte et al*.*^18^ evaluated the root canal morphology using cone-beam computed tomography (CBCT). In this context, a recent study established that CBCT is useful for detecting the configuration of the root canal in the mandibular first premolars; however, the obtained image details could be of lower quality, as compared with micro-CT^17^. Thus, some anatomic details could be left undetected when using CBCT as compared with micro-CT analysis^17^.

**Figure 6:**
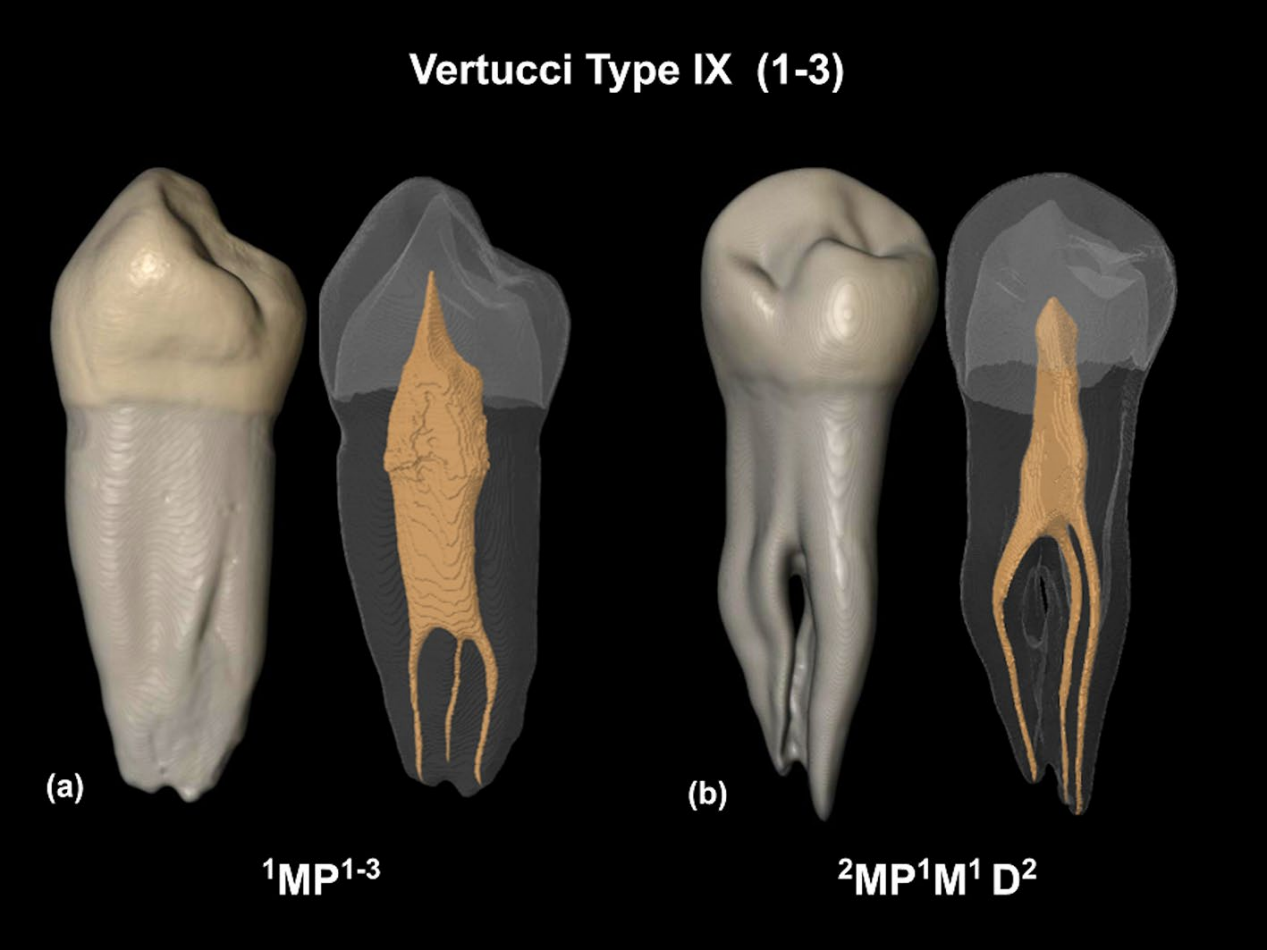
Micro-CT images showing the root canal configuration Vertucci’s type IX (1–3) in two teeth differently classified using Ahmed’s criteria. (**a**) ^1^MP^1–3^: A single-rooted mandibular first premolar with three root canals, (**b**) ^2^MP ^1^M^1^D^2^: A double-rooted mandibular first premolar with three root canals. *D* distal; *M* mesial; *MP* mandibular first premolar (Images obtained using the CTAn v.1.12 software, Bruker).

In the current study, Ahmed’s classification allowed the identification of seven different root canal configurations, being the code ^1^MP^1^ the most common in 65.05% of analyzed teeth, followed by the code ^1^MP^1–2^ in 24.19% of teeth. A different number of canal configurations were observed between Vertucci’s and Ahmed’s criteria, and this is because Ahmed’s classification considers the number of roots for coding. Indeed, two of the analyzed teeth were identified as Vertucci’s supplemental configuration IX (1–3); however, when Ahmed’s criteria were applied, one tooth was classified as code ^1^MP^1–3^, having a single root with three canals (Fig. 6a), and the other tooth was identified with the code ^2^MP ^1^M^1^D^2^, having two roots with three canals (one canal in the mesial root and two canals in the distal root) (Fig. 6b). In previous studies, mandibular first premolars with similar canal configuration types but with different root numbers were classified under the same type using Vertucci’s criteria^8,23^. Thus, mandibular first premolars with one or multiple roots and three root canals were all identified as Vertucci’s type VIII^8^, and maxillary premolars with one or two roots and two separate canals were all identified as Vertucci’s type VI^23^. In this sense, Ahmed’s coding system has clear advantages over Vertucci’s classification, due to the fact that it provides systematic and accurate information on the internal and external configuration of the tooth and more significant discrimination, especially in a group of teeth with one, two, or more roots. Indeed, Vertucci’s criteria may not be able to classify very complex configurations, which can be classified by Ahmed’s criteria.

The occurrence of root grooves had not been described in any teeth in the Chilean population. In the present study, 39.25% of the analyzed mandibular first premolars presented root grooves, similar to the incidence previously reported in Chinese populations with 40.9%^7^ and 44.38%^8^ of teeth. However, our results differ from those reported in the Brazilian population, with 14% of analyzed teeth^24^. The presence of deep radicular grooves in mandibular first premolars has been closely related to the detection of complex internal anatomy^7–9,15^. Dou et al*.*^8^ reported the detection of a multiple root canal system in 70% of teeth with shallow grooves (ASUDAS grade 1), 86.36% of teeth with moderately deep grooves (ASUDAS grade 2), and 100% of teeth with severe deep grooves (ASUDAS grades 3 and 4). In this study, a multiple root canal system was identified in 42.31% of teeth with shallow grooves (ASUDAS grade 1), 95.65% of teeth with moderately deep grooves (ASUDAS grade 2), and 100% of teeth with severe deep grooves (ASUDAS grades 3, 4, and 5). These results demonstrated a direct relationship between the severity of the radicular grooves and the detection of complicated root canal morphology in the mandibular first premolars of the Chilean population.

The accessory canals and apical deltas are the most complex structures in the root canal system and a considerable challenge to clean, disinfect, and seal during endodontic treatment. Indeed, the presence of an accessory canal is frequently associated with endo-periodontal lesions^25^. In this study, accessory canals were present in 62.90% of teeth, with most of them located at the apical third of the root canal, in a similar way to what has been previously reported^8^. Nevertheless, the apical delta was identified with a higher incidence in this study (26.88%) as compared with other studies reporting an incidence of 6.1%^5^ and 10.11%^8^ in Chinese populations, and 15.5%^21^ and 16.9%^26^ in Turkish populations. Besides, a high presence of multiple foramines was identified in this study (63.44%), which could be related to the presence of accessory canals and apical deltas in the apical third of the root canal.

The unpredictable location and anatomic complexity of isthmus make them difficult to clean and disinfect properly, representing a significant challenge to endodontists^27^. Indeed, an isthmus may contain necrotic debris, tissue remnants, or organic substrates that favor bacterial colonization^28^. In this study, isthmuses were identified in 31.72% of analyzed teeth, with most of them located at the middle third of the root canal, which represents a higher incidence than that reported in other studies also using micro-CT^5^. Conversely, a similar incidence to other reports was found for the lateral location of the apical foramen^5,8^, which was 62.37% in this study. On the other hand, the incidence of C-shaped canals in mandibular first premolars has been reported in other populations with variable frequency, ranging from 12.36%^8^ to 24%^14^. Compared to those studies, herein, a higher incidence of C-shaped canals was observed, being identified in 29.57% of the analyzed teeth.

The variability in the external and internal anatomy of the mandibular first premolar observed between the studies can be attributed to the genetic determinants, ethnicity, and geographical origin of the analyzed population^1,2^. From a clinical perspective, the wide range of anatomical variations of the mandibular first premolar described in this study in the Chilean population represents a remarkable task to endodontists. Indeed, a major reason for endodontic treatment failure is the inability to locate, debride, shape, and obturate a second or third canal in teeth with multiple-canal system^29^. Besides, C-shaped canals, isthmus, and accessory canals have an irregular and complex morphology, which is not compatible with the preparations provided by rotary instrumentation systems and frequently requires the use of complementary irrigation systems and intracanal medication^30,31^. In this study, the analysis using micro-CT allowed us to obtain a detailed description of the root canal system morphology of the mandibular first premolar, using Vertucci’s, Ahmed’s, and ASUDAS classifications. The data reported in this study may provide endodontists a more comprehensive understanding of the anatomy of this tooth, contributing to the achievement of successful endodontic treatments.

## Conclusions

The root canal system morphology of mandibular first premolars showed a wide range of anatomical variations in the Chilean population. Ahmed’s criteria allowed us to classify the internal anatomy of the root canal in a more precise and practical way than Vertucci’s criteria. According to Ahmed’s criteria, the code ^1^MP^1^ was the most prevalent among the analyzed teeth. Teeth with multiple root canals had a higher incidence of radicular grooves and a more complex morphology compared with teeth with a single root canal. Besides, the presence of radicular grooves was closely related to a more complex internal anatomy, and only teeth with multiple root canals presented Tomes’ anomalous root. Therefore, there is a close link between the external anatomical characteristics and internal complexity of the root canal system in the mandibular first premolars.

## References

[1] Trope M, Elfenbein L, Tronstad L (1986). Mandibular premolars with more than one root canal in different race groups. J. Endod..

[2] Cleghorn BM, Christie WH, Dong CC (2007). The root and root canal morphology of the human mandibular first premolar: a literature review. J. Endod..

[3] Vertucci FJ (2005). Root canal morphology and its relationship to endodontic procedures. Endod. Top..

[4] Cantatore G, Berutti E, Castellucci A (2006). Missed anatomy: frequency and clinical impact. Endod. Top..

[5] Liu N (2013). A micro-computed tomography study of the root canal morphology of the mandibular first premolar in a population from southwestern China. Clin. Oral Invest..

[6] Ordinola-Zapata R (2013). Morphologic micro-computed tomography analysis of mandibular premolars with three root canals. J. Endod..

[7] Chen J (2015). A micro-computed tomography study of the relationship between radicular grooves and root canal morphology in mandibular first premolars. Clin. Oral Invest..

[8] Dou L, Li D, Xu T, Tang Y, Yang D (2017). Root anatomy and canal morphology of mandibular first premolars in a Chinese population. Sci. Rep..

[9] Guerreiro D, Shin JM, Pereira M, McDonald NJ (2019). Radicular groove accessory canal morphology in mandibular first premolars: micro-computed tomographic study. J. Endod..

[10] Vertucci FJ (1978). Root canal morphology of mandibular premolars. J. Am. Dent. Assoc..

[11] Ahmed HMA, Versiani MA, De-Deus G, Dummer PMH (2017). A new system for classifying root and root canal morphology. Int.. Endod. J..

[12] Ahmed HMA, Neelakantan P, Dummer PMH (2018). A new system for classifying accessory canal morphology. Int. Endod. J..

[13] Ahmed HMA, Dummer PMH (2018). A new system for classifying tooth, root and canal anomalies. Int. Endod. J..

[14] Fan B, Yang J, Gutmann JL, Fan M (2008). Root canal systems in mandibular first premolars with C-shaped root configurations. Part I: Microcomputed tomography mapping of the radicular groove and associated root canal cross-sections. J. Endod..

[15] Gu Y, Zhang Y, Liao Z (2013). Root and canal morphology of mandibular first premolars with radicular grooves. Arch. Oral Biol..

[16] Weng XL (2009). Root canal morphology of permanent maxillary teeth in the Han nationality in Chinese Guanzhong area: a new modified root canal staining technique. J. Endod..

[17] Zhang D (2017). The root canal morphology in mandibular first premolars: a comparative evaluation of cone-beam computed tomography and micro-computed tomography. Clin. Oral Invest..

[18] Pedemonte E (2018). Root and canal morphology of mandibular premolars using cone-beam computed tomography in a Chilean and Belgian subpopulation: a cross-sectional study. Oral Radiol..

[19] Grande NM (2012). Present and future in the use of micro-CT scanner 3D analysis for the study of dental and root canal morphology. Ann. Ist Super Sanita..

[20] Mazzi-Chaves JF (2020). Micro-computed tomographic assessment of the variability and morphological features of root canal system and their ramifications. J. Appl.. Oral Sci..

[21] Sert S, Bayirli GS (2004). Evaluation of the root canal configurations of the mandibular and maxillary permanent teeth by gender in the Turkish population. J Endod..

[22] Alkaabi W, AlShwaimi E, Farooq I, Goodis HE, Chogle SM (2017). A Micro-computed tomography study of the root canal morphology of mandibular first premolars in an Emirati population. Med. Prin. Pract..

[23] Saber S, Ahmed MHM, Obeid M, Ahmed HMA (2019). Root and canal morphology of maxillary premolar teeth in an Egyptian subpopulation using two classification systems: a cone beam computed tomography study. Int. Endod. J..

[24] Boschetti E (2017). Micro-CT Evaluation of root and canal morphology of mandibular first premolars with radicular grooves. Braz. Dent. J..

[25] Ricucci D, Siqueira JF (2010). Fate of the tissue in lateral canals and apical ramifications in response to pathologic conditions and treatment procedures. J.. Endod..

[26] Calişkan MK, Pehlivan Y, Sepetçioğlu F, Türkün M, Tuncer SS (1995). Root canal morphology of human permanent teeth in a Turkish population. J.. Endod..

[27] Estrela C (2015). Frequency of root canal isthmi in human permanent teeth determined by cone-beam computed tomography. J. Endod..

[28] Carr GB, Schwartz RS, Schaudinn C, Gorur A, Costerton JW (2009). Ultrastructural examination of failed molar retreatment with secondary apical periodontitis: an examination of endodontic biofilms in an endodontic retreatment failure. J. Endod..

[29] Costa F (2019). Association between missed canals and apical periodontitis. Int. Endod. J..

[30] Urban K, Donnermeyer D, Schäfer E, Bürklein S (2017). Canal cleanliness using different irrigation activation systems: a SEM evaluation. Clin.. Oral Invest..

[31] Siqueira Junior JF, Rôças IDN, Marceliano-Alves MF, Pérez AR, Ricucci D (2018). Unprepared root canal surface areas: causes, clinical implications, and therapeutic strategies. Braz. Oral Res..

